# Some Immunohormonal Changes in Experimentally Pregnant Toxemic Goats

**DOI:** 10.4061/2010/768438

**Published:** 2010-06-16

**Authors:** Abd-Elghany Hefnawy, Seham Youssef, Saad Shousha

**Affiliations:** ^1^Department of Internal Medicine, Faculty of Veterinary Medicine, Benha University, Moshtohor 13736, Egypt; ^2^Department of Pharmacology, Faculty of Veterinary Medicine, Benha University, Moshtohor 13736, Egypt; ^3^Department of Physiology, Faculty of Veterinary Medicine, Benha University, Moshtohor 13736, Egypt

## Abstract

Pregnancy toxemia was induced in nine pregnant goat does with twins by the stress of fasting with access to water in late pregnancy to investigate the effect of pregnancy toxemia on immunoglobulins (IgA, IgM, and IgG), cortisol, insulin, thyroid, and growth hormones and their correlations with the plasma levels of glucose and *β*-Hydroxybutyrate. Plasma samples were collected at 0, 12, 24, 36, 48, and 72 hours after induction of pregnancy toxemia. The result revealed that experimental animals developed neurological findings with convulsions and acetone odor from the mouth with recumbency after 72 hours. Laboratory findings showed a significant increase in *β*-Hydroxybutyrate, cortisol, and insulin while there were significant decreases in glucose, thyroid, and immunoglobulins (IgA, IgM, and IgG). Plasma glucose concentrations had significant negative correlations with *β*-hydroxybutyrate, cortisol, and insulin while the correlations were significantly positive with immunoglobulins and thyroid hormone. Plasma *β*-hydroxybutyrate concentration was significantly positively correlated with cortisol and negatively correlated with immunoglobulins, insulin, and thyroid hormone. From this study we can conclude that pregnancy toxemia might affect humoral immune responses as well as insulin, cortisol, and thyroid hormones. Moreover, insulin might have a compensatory role to increase suppressive effect on ketogenesis in experimentally pregnant toxemic goats.

## 1. Introduction

Pregnancy represents one of the most anabolic periods of the female life cycle, and pregnancy toxemia is a disease caused by a negative energy balance in late gestation and seen commonly in ewes, guinea pigs, rabbits, and occasionally in cows, ferrets, sows, and many other species. Predisposing causes include stress and food deprivation or negative energy balance in late pregnancy [1–5]. Pregnancy toxemia frequently develops during the last 4 to 6 weeks of gestation in sheep and goat, primarily in pregnancies with more than one fetus. About 60% of fetal growth takes place in this last gestation period [6], and during this time approximately 33%–36% of the circulating glucose is directed into fetoplacental unit to satisfy its energetic demands [7].

The endocrine system especially the pancreas probably is intimately involved in the development of ruminant ketosis [8]. Insulin inhibits ketogenesis when free fatty acids levels are high [9], as well as growth hormone secretions inhibited by cortisol and free fatty acids [10]. Insulin also appears to be important in regulating the utilization of ketone bodies as the uptake of *β*-hydroxybutyrate and acetate by sheep hind limbs is impaired during alloxan diabetes and restored by insulin [11]. Major changes in blood are associated with development of clinical ketosis [12, 13]. 

The principal blood changes in pregnancy toxemia are increase in plasma *β*-hydroxyl butyrate and decrease in plasma glucose [14]. Ketosis is interrelated with several infectious diseases of dairy cattle [15, 16]. These reports provide justification for studying possible interactions between immunoglobulins and various metabolites that are characteristic of pregnancy toxemia in goats as well as its effect on some hormones. The purpose of this study was to elucidate the effect of experimental pregnancy toxemia on some immunological and hormonal parameters in goats.

## 2. Material and Methods

 Nine pregnant goat does with twins of 3-4 years old, 20–27 Kg body weight, and 120–130-days of gestation were used for induction of pregnancy toxemia by fasting for 72 hours until the symptoms of pregnancy toxemia appeared. All investigated animals were fed on 250 grams corn/head/day, concentrates, and berseem ad lib for two weeks before the beginning of the experiment, and NIH guidelines for the care and use of animals have been followed. Serum samples were collected at 0, 12, 24, 36, 48, and 72 hours from the beginning of the induction of pregnancy toxemia and stored at +4°C (≤ 48 h) until assay of *β*-hydroxybutyrate [17] and glucose concentrations or at −20°C until insulin, T4, T3, cortisol, and growth hormone concentrations were determined by radioimmunoassay (RIA) and immunoglobulins levels according to the method described in [18]. 

## 3. Statistical Analysis

 For presentation of results, the means and their standard errors (SEM) were calculated. The results were subjected to student's *t*-test. Pearson correlation coefficient by general linear model and regression analyses was performed using the Statistical Analysis System software [19]. Results were considered statistically significant when *P* < .05.

## 4. Results

**Figure 1:**
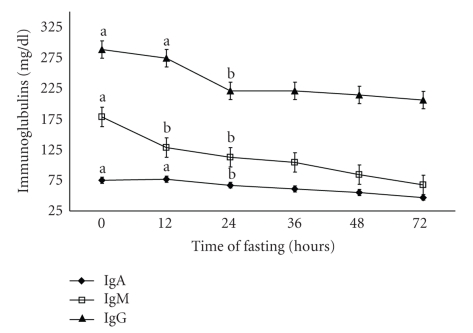
Concentration of immunoglobulins (IgA, IgM, and IgG) mg/dl in experimentally pregnant toxemic goats. Differences in the letters indicate beginning of the significant differences.

**Figure 2:**
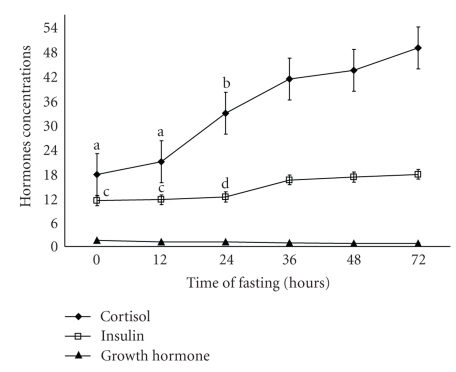
Concentration of Cortisol (*μ*g/dl), Insulin hormone (*μ*Iu/ml) and Growth hormone in experimentally pregnancy toxemic goats. Differences in the letters indicate beginning of the significant differences.

Clinical examination of the animals revealed that the clinical symptoms of pregnancy toxemia appeared after 72 hours in the form of dullness, ruminal stasis, grinding on the teeth, and abnormal pasture as listening attitude, stargazing position, and lateral recumbence with acetone odor of the mouth, urine and general weakness with pale mucous membrane. In this study, there were significant (*P* < .01) decreases in IgA, IgM, and IgG of pregnant toxemic goat does at 24 h, 12 h, and 24 h, respectively, after induction of pregnancy toxemia (Figure 1). There were significant (*P* < .01) increase in both cortisol and insulin in pregnancy toxemic animals at 24 h and 36 h, respectively, after induction of pregnancy toxemia (Figure 2).

**Figure 3:**
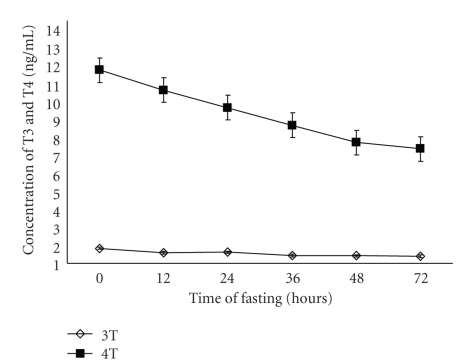
Concentration of T3 and T4 (ng/ml) in experimentally pregnant toxemic goats. Differences in the letters indicate beginning of the significant differences.

**Figure 4:**
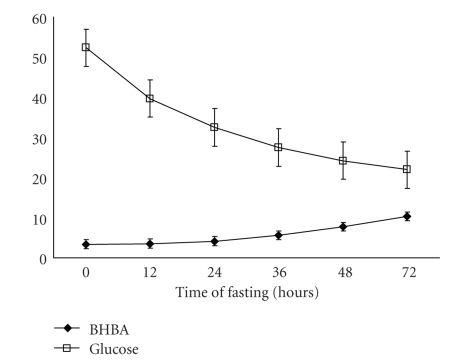
Concentration of *β*-HBA and glucose (mg/dl) in experimentally pregnancy toxemic goats. Differences in the letters indicate beginning of the significant differences.

 There was a significant (*P* < .01) decrease in T_4_ in pregnancy toxemic animals at 24 h after induction of pregnancy toxemia, while there were no significant changes in both growth hormone and T_3_ along the time of experiment (Figures 2 and 3). The concentration of *β*-hydroxybutyrate was significantly (*P* < .01) increased at 36 h of induction of pregnancy toxemia while glucose concentration was significantly (*P* < .01) decreased at 24 h of induction of pregnancy toxemia (Figure 4).

There were significant negative relationships between glucose concentrations and cortisol, insulin and *β*-hydroxybutyrate, while the relationships were significantly positive with IgA, IgM, IgG, growth hormone, T_3_,and T_4_. The relationships between *β*-hydroxybutyrate concentration and IgA, IgM, IgA, T_4_, and insulin were significantly negative, while the relationship with cortisol was significantly positive. (Table 1).

## 5. Discussion

The present study aimed to evaluate the effect of experimental pregnancy toxemia induced by short fasting treatment for 72 hours on immunoglobulins and some hormones in goats. The present study clarified a significant decrease in IgA, IgM, and IgG levels with significant positive correlations between glucose concentration and immunoglobulins. Also there were marked negative correlations between *β*-hydroxybutyrate and immunoglobulins in pregnancy toxemic goats. These data were in contrast with previous studies in [14] which indicated that effects of ketone and acetate concentrations associated with bovine ketosis did not alter IgM secretion in vivo [20] and did not detect any significant relationships between plasma indicators of metabolic condition (plasma glucose and acetoacetate) and immune functions (serum and milk IgG, total number of peripheral leukocytes) in dairy cows.

Ketone inhibits bovine leukocyte functions in vitro, and these results suggested that this effect might affect the in vivo immune response negatively [21, 22]. Ketone bodies at pathological concentrations are reported to reduce bovine T-lymphocytes blastogenesis [23]. Therefore, the immunosuppressive status of ketotic animals may be a result of alteration of specific and/or nonspecific immunity imputable to ketone bodies themselves [24]. ketone bodies in particular *β*-hydroxybutyrate are able to depress in vitro two steps of phagocytic process at concentration similar to that observed during ketosis in sheep [25] and affect IgG [26].

The significant increase in cortisol and presence of significant negative correlation between plasma glucose concentration and cortisol level and the significant positive relationship with *β*-hydroxybutyrate may be due to increased adrenal output or to impaired ability of the fatty liver, which was a consistent finding in pregnancy toxemia (unpublished data), to mobilize and excrete the hormone [27]. It is indicated that the concentration of glucose in plasma was below and *β*-hydroxybutyrate (the major ketone body of ruminants) was above the normal range during pregnancy toxemia, and there was a significant negative correlation between ketone bodies and glucose [28]. Also, it is recorded that, there was a significant positive correlation between *β*-hydroxybutyrate and cortisol in subclinical pregnancy toxemic goat does [29].

The significant decrease in T_4_ in pregnancy toxemic goats may be attributed to excessive secretion of cortisol as there is a negative correlation between free T_4_ and cortisol as concluded in [30]. The response to fasting (negative energy balance) incorporates hormonal signals which initiate energy preservation. Insulin, T_4_, and T_3_ are important hormones in the regulation of energy homeostasis. The decreases in T_4_ in experimental pregnancy toxemic goats in the present study were similar to that recorded in ewes [5] and ferret [31] with pregnancy toxemia.

It is well known that insulin alters fatty acid release and also alters ketogenesis. Furthermore, insulin appears to suppress ketogenesis independent of any effect of free fatty acids concentrations [32]. Insulin appears to be important in regulating the utilization of ketone bodies, and the uptake of *β*-hydroxybutyrate and acetate by sheep hind limbs is impaired during alloxan diabetes and is restored by insulin [11]. Insulin increased the rate of removal of ketone bodies from blood, and during insulin deficiency maximal utilization of ketone bodies was impaired [33]. Moreover, insulin deficiency increased lipolysis and increased production of ketone bodies [10]. The significant increase of insulin concentration in our study may be considered as a compensatory role for increasing the suppressive effect of insulin on the ketogenesis and impairs production of ketone bodies. These results are in contrary to those in [5, 32] that pregnancy toxemia associated with hypoinsulinemia.

From this study we can conclude that pregnancy toxemia has immunosuppressive effect in vivo as well as it alters concentrations of insulin, cortisol, and thyroid hormone that correlated with the levels of plasma glucose and *β*-hydroxybutyrate, and insulin may play a metabolic adaptive role to suppress ketogenesis and decrease ketone bodies production in experimentally pregnant toxemic goats.

## Figures and Tables

**Table 1:** The correlations of glucose and *β*-HBA concentrations with immunoglobulins, cortisol, insulin, growth hormone, and thyroid hormones in pregnancy toxemic goats.

	Glucose	*β*-HBA
IgA	0.87*	−0.96**
IgM	0.90**	−0.84*
IgG	0.95*	−0.75
Cortisol	−0.90**	0.88*
Insulin	−0.87*	−0.91*
Growth hormone	0.97***	NS
T3	0.96***	NS
T4	0.97***	−0.90*
Glucose	—	−0.80*

*P < .5*P < .01***P < .001.
